# Supplementary material for: Genome-wide investigation of gene-cancer associations for the prediction of novel therapeutic targets in oncology
Source: Sci Rep. 2020 Jul 1;10:10787. doi: 10.1038/s41598-020-67846-1 (PMC7330039; doi:10.1038/s41598-020-67846-1)
Supplement: Supplementary file 1 — Supplementary material 1 (pdf 161 KB) [file 41598_2020_67846_MOESM1_ESM.pdf]

Supplementary Material for:

Genome-wide investigation of gene-cancer associations for the prediction of novel therapeutic targets in oncology

by

Adrián Bazaga, Dan Leggate and Hendrik Weisser

Supplementary Table S1: Details of the artificial neural network architecture used in this work.

Layer	Type	Number of neurons (output)	Activation function
1	Fully-connected	64	ReLU
2	Fully-connected	128	ReLU
3	Fully-connected	16	ReLU
4	Fully-connected	2	Sigmoid

Supplementary Table S2: Hyperparameter search space for each of the machine learning methods

Method	Parameters and range of values
Random Forest	Max depth = [4, 5, 6, 7, 8]<br># estimators = [300, 500, 700, 850, 1000]<br>Max features = [sqrt(# features), log2(# features), 30%, 50%]
Support Vector Machine	Kernel function = [linear, rbf].<br>For RBF kernel, gamma = [1e-3, 1e-4], C = [1, 10, 100, 1000].<br>For linear kernel, C = [1, 10, 100, 1000]
Gradient Boosting Machine	Learning rate = [0.005, 0.1].<br>Max depth = [4, 5, 6, 7, 8].<br># estimators = [300, 500, 700, 850, 1000].<br>Max features = [sqrt(# features), log2(# features), 30%, 50%]
Logistic Regression	N/A

Supplementary Table S3: Performance in terms of test set AUC achieved by each of the five different machine learning methods across cancer types.

Cancer type / Method	Bladder	Breast	Colon	Kidney	Leukemia	Liver	Lung	Ovarian	Pancreatic
Logistic Regression	0.78	0.77	0.69	0.86	0.75	0.84	0.81	0.79	0.75
Support Vector Machine	0.77	0.78	0.72	0.88	0.72	0.84	0.87	0.8	0.73
Gradient Boosting Machine	0.75	0.7	0.74	0.75	0.71	0.81	0.73	0.74	0.73
Neural Network	0.67	0.72	0.71	0.71	0.7	0.86	0.75	0.75	0.72
Random Forests	0.76	0.75	0.76	0.79	0.74	0.85	0.83	0.77	0.76

Supplementary Table S4: Total number of genes predicted as targets (probability ≥ 0.5) by the best model for each of the cancer types

Cancer type	Number of predicted targets
Bladder	4473/15500 (28%)
Breast	4129/15500 (26%)
Colon	3246/15500 (20%)
Kidney	5451/15500 (35%)
Leukemia	4272/13600 (31%)
Liver	3188/15500 (20%)
Lung	4502/15500 (29%)
Ovarian	4681/15500 (30%)
Pancreatic	3750/15500 (24%)

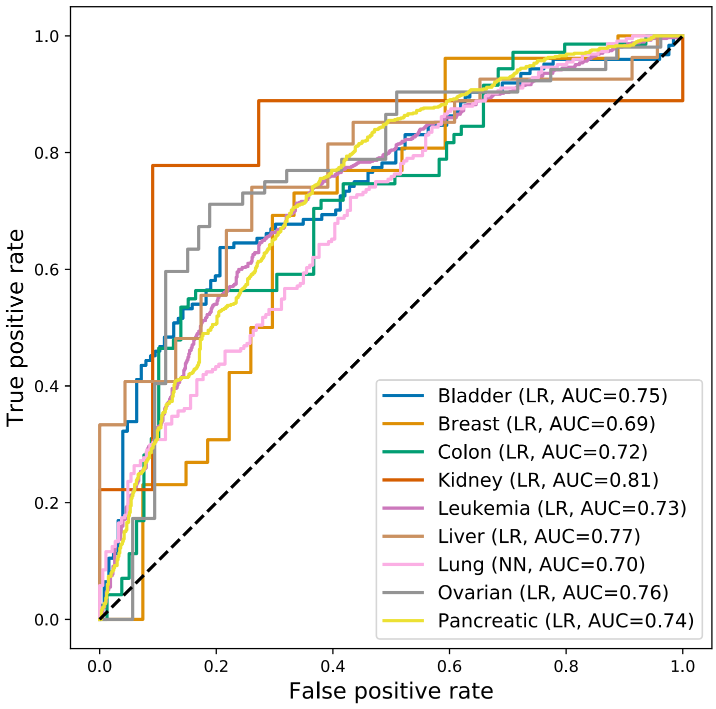

Supplementary Figure S1: Generalization performances on the test sets for the best models across cancer types, measured in terms of AUROC, using only the network embedding features.

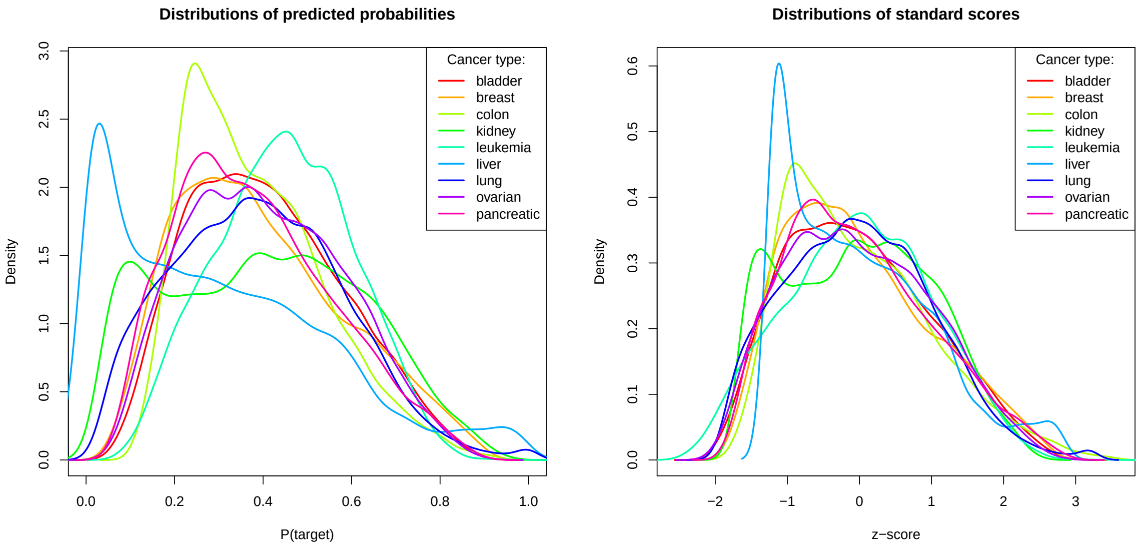

Supplementary Figure S2: Distributions (kernel density estimates) of genome-wide predicted probabilities for different cancer types, before (left) and after (right) scaling per cancer type.

Supplementary Table S5: Top 5 predictions for ovarian cancer.

Gene	Full name	Probability	Citations
SLA	Src Like Adaptor	0.919	0
LY6E	Lymphocyte Antigen 6 Family Member E	0.914	0
TYROBP	TYRO Protein Tyrosine Kinase Binding Protein	0.909	0
JAK1	Janus Kinase 1	0.908	0
CCDC74A	Coiled-Coil Domain Containing 74A	0.905	0

Supplementary Table S6: Top 5 predictions for pancreatic cancer.

Gene	Full name	Probability	Citations
STAT1	Signal Transducer And Activator Of Transcription 1	0.909	2
PTPN12	Protein Tyrosine Phosphatase Non-Receptor Type 12	0.901	1
MYO1D	Myogenic Differentiation 1	0.899	1
NBEAL2	Neurobeachin Like 2	0.896	0
INTS3	Integrator Complex Subunit 3	0.895	1

Supplementary Table S7: Top 5 predictions for kidney cancer.

Gene	Full name	Probability	Citations
TYROBP	TYRO Protein Tyrosine Kinase Binding Protein	0.961	0
SLA	Src Like Adaptor	0.958	0
PEAR1	Platelet Endothelial Aggregation Receptor 1	0.956	0
JAK1	Janus Kinase 1	0.952	0
KL	Klotho	0.950	0

Supplementary Table S8: Top 5 predictions for bladder cancer.

Gene	Full name	Probability	Citations
MDFIC	MyoD Family Inhibitor Domain Containing	0.930	0
PRDM2	PR/SET Domain 2	0.908	0
POU3F1	POU Class 3 Homeobox 1	0.904	3
HMGA1	High Mobility Group AT-Hook 1	0.888	0
PRSS8	Serine Protease 8	0.887	2

Supplementary Table S9: Top 5 predictions for liver cancer.

Gene	Full name	Probability	Citations
PEAR1	Platelet Endothelial Aggregation Receptor 1	0.999	0
SLA	Src Like Adaptor	0.999	0
SIT1	Signaling Threshold Regulating Transmembrane Adaptor 1	0.998	2
ZAP70	Zeta Chain Of T Cell Receptor Associated Protein Kinase 70	0.998	0
FCRL3	Fc Receptor Like 3	0.998	0

Supplementary Table S10: Top 5 predictions for lung cancer.

Gene	Full name	Probability	Citations
TENM1	Teneurin Transmembrane Protein 1	1.0	0
CCDC7	Coiled-Coil Domain Containing 7	1.0	0
NAA38	N(Alpha)-Acetyltransferase 38, NatC Auxiliary Subunit	1.0	0
B3GNT2	UDP-GlcNAc:BetaGal Beta-1,3-N-Acetylglucosaminyltransferase 2	1.0	0
RPS6KA2	Ribosomal Protein S6 Kinase A2	1.0	1
